# The Influence of Acute Cold Stress on Intestinal Health of the Juvenile Chinese Soft-Shelled Turtle (*Pelodiscus sinensis*)

**DOI:** 10.3390/ani16020256

**Published:** 2026-01-14

**Authors:** Xiaona Ma, Qing Shi, Zhen Dong, Chen Chen, Junxian Zhu, Xiaoli Liu, Xiaoyou Hong, Chengqing Wei, Xinping Zhu, Weijia Song, Wei Li, Liqin Ji

**Affiliations:** 1 Jiangsu Key Laboratory of Marine Biotechnology, Jiangsu Ocean University, Lianyungang 222005, China; marianna_iocas@163.com (X.M.); sq1014yl@126.com (Q.S.); songwj178@163.com (W.S.); 2 Key Laboratory of Tropical and Subtropical Fishery Resources Application and Cultivation, Ministry of Agriculture and Rural Affairs, Pearl River Fisheries Research Institute, Chinese Academy of Fishery Sciences, Guangzhou 510380, China; chenchen@prfri.ac.cn (C.C.); zjzhujunxian@prfri.ac.cn (J.Z.); liuxl@prfri.ac.cn (X.L.); hxy@prfri.ac.cn (X.H.); zjweichengqing@prfri.ac.cn (C.W.); zhuxinping@prfri.ac.cn (X.Z.); 3 Key Laboratory of Marine Environmental Survey Technology and Application, South China Sea Marine Survey Center, Ministry of Natural Resources, Guangzhou 510275, China; dongzhen@stu.ouc.edu.cn

**Keywords:** cold stress, intestine, histology, microbial communities, multi-omics, Chinese soft-shelled turtle

## Abstract

Sharp declines in temperature pose a significant risk for mass mortality events in the Chinese soft-shelled turtle (*Pelodiscus sinensis*). To assess the effects of acute cold stress on intestinal health, turtles were exposed to temperatures of 28 °C (control), 14 °C, and 7 °C for 1, 2, 4, 8, and 16 days. The results showed that acute cold stress at 14 °C and 7 °C induced time-dependent alterations in intestinal morphology and histopathology. The damage was more severe at 7 °C, characterized by inflammatory cell infiltration, lymphoid hyperplasia, and extensive detachment and necrosis across the villi, muscle layer, and submucosa. 16S rDNA sequencing revealed significant shifts in intestinal microbiota composition in the 7 °C group, dominated by *Helicobacter* and *Citrobacter*. Transcriptomic analysis identified differentially expressed genes (DEGs) that respond to acute cold stress and are involved in the Toll-like receptor signaling pathway (*Tlr2*, *Tlr4*, *Tlr5*, *Tlr7,* and *Tlr8*), the NOD-like receptor signaling pathway (*Traf6, Traf2*, *Casr*, *Rnasel*, *Pstpip1*, *Plcb2*, *Atg5*, and *Mfn2*), apoptosis (*Tuba1c*, *Ctsz*, *Ctsb*, *Kras*, *Hras*, *Pik3ca*, *Bcl2l11*, *Gadd45a*, *Pmaip1*, *Ddit3*, and *Fos*), and the p53 signaling pathway (*Serpine1*, *Sesn2*, *Ccng2*, *Igf1*, *Mdm2*, *Gadd45a*, *Pmaip1*, and *Cdkn1a*). Metabolomic profiling highlighted differentially expressed metabolites (DEMs) that cope with acute cold stress, such as organic acids (oxoglutaric acid, L-aspartic acid, fumaric acid, DL-malic acid, and citric acid) and amino acids (including L-lysine, L-homoserine, and allysine). The integrated analysis of DEGs and DEMs underscored three key pathways modulated by acute cold stress: linoleic acid metabolism, neuroactive ligand–receptor interaction, and the FoxO signaling pathway. This study provides a comprehensive evaluation of intestinal health in Chinese soft-shelled turtles under acute cold stress and elucidates the underlying mechanisms.

## Simple Summary

As a poikilothermic species, the Chinese soft-shelled turtle is highly sensitive to changes in environmental temperature. Sudden drops in temperature often lead to mass mortality, causing substantial economic losses to the aquaculture industry. This study aims to uncover the mechanisms of damage induced by low-temperature stress in the Chinese soft-shelled turtle. Through integrated analysis of intestinal microbiota, molecular expression, metabolite profiles, and related signaling pathways, we further elucidate the physiological and molecular responses of this species to cold stress, providing a scientific basis for understanding its low-temperature adaptability.

## 1. Introduction

The Chinese soft-shelled turtle (*Pelodiscus sinensis*), as a member of the class Reptilia, order Testudines, and family Trionychidae, is a traditional and economically important aquaculture species in Southeast Asia. It is popular in Chinese markets for its high protein content and nutritional value, particularly its richness in bioactive peptides [1]. By 2023, national production of the Chinese soft-shelled turtle had reached nearly 0.5 million tons [2]. As an ectotherm with an optimal temperature range of 25–32 °C, this species is highly susceptible to water temperature fluctuations, which can cause mass mortality [3]. However, the adverse effects of acute cold stress on the Chinese soft-shelled turtle remain less comprehensively studied in comparison with other aquatic species.

Given the increasing frequency of extreme low-temperature events occurring from climate change and seasonal variation [4], the impact of cold stress on aquatic animals has been widely studied, especially in fishes [5]. A drastic reduction in temperature can profoundly affect physiological processes, metabolic traits, and immune response [6]. For instance, cold stress can alter biochemical parameters, cause DNA damage, and interfere with immune processes in the orange spotted grouper [7]. Research on *Takifugu fasciatus* has revealed that cold exposure leads to hepatic structural damage, fibrosis, oxidative stress, and lipid peroxidation [8]. Further research on hybrid sturgeon has found that acute cold stress suppresses the immune response by regulating apoptosis and the Toll-like receptor signaling pathway [9]. In contrast, the influence of low temperature on the Chinese soft-shelled turtle remains less well understood [10]. Therefore, investigating the adverse effects of cold stress is crucial for developing effective strategies to mitigate cold-stress-induced damage or death.

Seasonal cooling is a slow and gradual process, providing aquatic animals sufficient time to mobilize physiological compensation mechanisms—including the regulation of metabolic pathways and gene expression—for successful cold adaptation [5]. Conversely, acute cold stress occurs when water temperatures decline within hours, eliciting intensive physiological responses and exerting oxidative stress [9]. Although seasonal temperature variation can influence aquatic health, it typically enhances long-term survival through physiological acclimation. However, acute cold stress commonly surpasses an organism’s capacity to maintain homeostasis, leading to behavioral imbalances, incapacitating shock, and high mortality rates [11].

The intestine is a vital organ responsible for nutrient absorption and immune response [12]. Its physiological functions are highly sensitive to environmental stimuli [13], particularly temperature variations [14]. Studies on largemouth bass have found that an acute decrease in temperature alters gut morphology, characterized by increased villus length and reduced goblet cell numbers [15]. Temperature fluctuation leads to intestinal dysbiosis, subsequently leading to metabolic disorders and impaired immune function [16,17]. Similarly, research on tsinling lenok trout shows that heat stress results in gut microbial disturbance and metabolic dysregulation [14].

Our previous work revealed that acute cold stress triggers energy metabolic disorders, tissue damage, and apoptosis in the liver of the Chinese soft-shelled turtle [18]. The liver as the metabolic center and the intestine as the primary immune and barrier organ are functionally linked via the gut–liver axis, which facilitates bidirectional functional communication. To understand the organism’s systemic response to acute cold stress, the present study was thus conducted to evaluate the effects on the intestine, aiming to elucidate the distinct and coordinated physiological responses in the two organs. Our findings will provide novel insights into the intestinal adaptive mechanisms against acute cold stress, while offering potentially practical strategies for maintaining intestinal homeostasis in the Chinese soft-shelled turtle under acute cold stress.

## 2. Materials and Methods

### 2.1. Animal Ethics

The animal trials in this research complied with Chinese guidelines for laboratory animal care and use. The animal trial procedures were approved by the Ethics Committee of the Pearl River Fisheries Research Institute, Chinese Academy of Fishery Sciences (Approval No. LAEC-PRFRI-2023-10-15).

### 2.2. Experimental Design

The Chinese soft-shelled turtles were kindly gifted by the Huizhou Wealth Xing Industrial Co., Ltd. (Huizhou, China). A total of 180 juvenile turtles, aged 3 months with an average weight of 12 ± 3 g, were randomly selected for this research. Sex could not be determined visually at this juvenile stage and was therefore not considered. All of the experimental turtles were in good health, displaying no signs of disease or abnormal behavior before the experiment. The trial was conducted in the Pearl River Fisheries Research Institute (Guangzhou, China). Before initiating the cold stress challenge, the turtles were acclimated in polyethylene boxes (1 m × 1 m × 0.25 m) for 14 days. During acclimation, they were fed commercial pellets gifted by Guangdong Nutriera Group Co., Ltd. (Guangzhou, China) twice daily at 9:00 and 17:00 until visibly satiated. The water quality parameters were maintained as follows: temperature 28 ± 1 °C, pH 8.2 ± 0.4, NO_2_^−^ 1.0 ± 0.3 mg/L, NH_4_^+^-N 1.1 ± 0.5 mg/L, and dissolved oxygen 5.8 ± 1.5 mg/L. One-third of the farming water was changed once a week to maintain stable water conditions.

After acclimation, the 180 Chinese soft-shelled turtles were randomly and evenly distributed into 18 polyethylene boxes (48 cm × 35 cm × 17 cm, 10 turtles per box). These boxes were randomly assigned to three groups with three replicates: a 28 °C control group (CG) and 14 °C and 7 °C acute cold stress groups (T14 and T7). After a 24 h starvation period, the turtles were transferred to RXZ-436 cooling incubators (Ningbo Jiangnan Instrument Factory, Ningbo, China) for the acute cold stress challenge. The water temperature in the incubators was gradually lowered from 28 °C to the target temperatures at a rate of 1 °C/h and then kept at 28 °C, 14 °C, and 7 °C for 1, 2, 4, 8, and 16 days.

### 2.3. Sample Collection

The beginning of the acute cold stress experiment was defined as the time point when the water reached the target temperature. Sampling was conducted at 1, 2, 4, 8, and 16 days post cold stress (dps) with 12 replicates at each time point for each group. Before sampling, the turtles were anesthetized by immersion in a 1 g/L solution of tricaine methanesulfonate (MS-222). Afterwards, the turtles were quickly dissected on ice to collect the intestine. The small intestine, defined as the proximal 65% of the total intestinal length (regions S1–S5, Supplementary Figure S1) [19], was chosen for its primary roles in nutrient absorption and immune function. After fixation in Bouin’s solution overnight, the intestinal tracts were washed three times (10 min each) with 70% ethanol to remove residual fixative. The remaining small intestinal tracts from 12 individuals per group were pooled into 6 tubes (intestines from two turtles within the same tank were pooled into one sample), snap-frozen in liquid nitrogen, and stored at −80 °C for subsequent analysis of the microbiome, transcriptome, and metabolome.

### 2.4. Intestinal Histological Analysis

After overnight fixation in Bouin’s solution, the intestinal tracts were washed with 70% ethanol three times (10 min each) to remove residual Bouin’s solution. The intestinal tracts were then dehydrated through a graded ethanol series (80%, 90%, 95%, and 100%), equilibrated with xylene, and embedded in paraffin. Tissue sections (4 μm) were prepared using a Leica RM 2016 microtome (Leica Biosystems Co., Ltd., Wetzlar, Germany), mounted on slides, dewaxed in xylene, and stained with hematoxylin and eosin (H&E). After staining, sections were sealed with resin, and intestinal morphology was observed and captured using a NIKON ECLIPSE 100 upright optical microscope (Nikon Corporation, Tokyo, Japan). The morphological parameters, including villus height, villus width, lamina propria width, submucosal width, and muscularis layer width, were measured using ImageJ 1.44 software (National Institute of Health, Bethesda, MD, USA). For histological analysis, three individuals per group (*n* = 3) were assessed at each time point. The value for each individual was derived from the mean of five measurements per parameter. Consequently, statistical analysis was performed using three mean values as independent data points (*n* = 3) for each group at each time point.

### 2.5. Intestinal Microbiota Analysis

Six mixed intestinal samples (*n* = 6) per group were analyzed for microbiota analysis, with each sample representing a blend of intestines from two turtles within the same tank. The Fast DNA SPIN kit (MP Biomedicals, Irvine, CA, USA) was utilized for microbial genomic DNA extraction. After assessing the DNA quality and concentration, the V3-V4 hypervariable region of the bacterial 16S rDNA gene was amplified using primers 338F: 5′-ACTCCTACGGGAGGCAGCA-3′ and 806R: 5′-GGACTACHVGGGTWTCTAAT-3′ with the diluted DNA as a template. PCR amplification was conducted in a final volume of 25 µL. The reaction mixture comprised 5 µL of 5 × Q5 Reaction Buffer, 5 µL of 5× Q5 High GC Buffer, 0.25 µL of Q5 High-Fidelity DNA Polymerase, 2 µL of dNTPs, 1 µL each of forward and reverse primers, 2 µL of template DNA, and nuclease-free water to volume. The PCR program was set as follows: an initial denaturation at 98 °C for 5 min, followed by 25 cycles of 98 °C for 30 s, 52 °C for 30 s, and 72 °C for 45 s, with a final extension at 72 °C for 5 min. All samples were run in triplicate, and a no-template control (NTC) was included for quality assurance. The PCR product was purified using the Omega DNA purification kit (Omega Inc., Norcross, GA, USA) and sequenced on the Illumina NovaSeq 6000 platform by Suzhou PANOMIX Biomedical Tech Co., Ltd., China. The final clean reads were obtained by removing chimeric sequences from raw data.

Sequences were assigned to the same operational taxonomic unit (OTU) when their similarity exceeded 97%, as implemented in the Uparse software (version 7.0.1001) [20]. Alpha diversity indices (Good’s coverage, Chao1, Observed species, Shannon, Simpson, Faith’s PD, and Pielou’s evenness), β diversity (principal coordinate analysis), and bacterial abundance at the phylum and genus levels were analyzed with QIIME2 software. The linear discriminant analysis (LDA) effect size (LEfSe) was employed to identify differentially abundant taxa between CG and T7. The potential function of the microbial communities was predicted from the 16S rDNA data using PICRUSt2. The resulting gene families were then annotated against the KEGG database (http://www.genome.jp/kegg/pathway.html, (accessed on 24 November 2024)). The abundances of KEGG pathways were compared between the CG and T7 groups to assess differences in metabolic functions.

### 2.6. Intestinal Transcriptomic Analysis

Small intestines from the CG and T7 groups at 8 days post stress (dps) were collected and subjected to transcriptomic analysis. Three pooled samples (*n* = 3), each representing a mix from four individuals in the same box, were analyzed. Total RNA was isolated from the intestine using TRIzol reagent (Invitrogen Life Technologies, Waltham, MA, USA). RNA integrity was initially assessed via electrophoresis on 1% agarose gels. RNA concentration and purity were determined using a NanoDrop 2000 spectrophotometer (Thermo Fisher Scientific, Waltham, MA, USA). Furthermore, the RNA Integrity Number (RIN) was precisely quantified with an Agilent 2100 Bioanalyzer (Agilent Technologies, Waltham, MA, USA). Only RNA samples with an RIN greater than 7 were used to construct the cDNA library.

Raw data were processed to remove adapters and low-quality reads. The clean data were subsequently mapped to the Chinese soft-shelled turtle genome (https://www.ncbi.nlm.nih.gov/datasets/genome/GCF_000230535.1/ (accessed on 24 July 2012)). Differentially expressed genes (DEGs) between CG and T7 were identified using the DESeq2 R package (v1.20.2), with screening thresholds set at |log_2_(fold change)| ≥ 1.0 and adjusted *p* < 0.05. Subsequently, these DEGs were functionally annotated using the Gene Ontology (GO) and Kyoto Encyclopedia of Genes and Genomes (KEGG) databases.

### 2.7. Intestinal Metabolomic Analysis

Six small intestines per group from CG and T7 at 8 dps were used for metabolomic analysis (*n* = 6). Metabolites were extracted from 100 mg of intestine with a 1 mL methanol/chloroform/water mixture (27:3:1, *v*/*v*/*v*), which was then homogenized, ultrasonicated, and centrifuged at 12,000 rpm for 10 min. After vacuum drying, the supernatant was redissolved in 400 µL of methanol-water (1:1) containing 4 ppm 2-chloro-L-phenylalanine as the LC-MS sample. Chromatographic separation was achieved on a Thermo Vanquish ultra-high-performance liquid chromatography system equipped with an ACQUITY UPLC^®^ HSS T3 column (Waters, Milford, MA, USA). System stability was monitored by analyzing a quality control (QC) sample, which was a pool of equal aliquots from all experimental samples, alongside the study samples using a Thermo Q Exactive HF-X mass spectrometer (Thermo Fisher Scientific, Waltham, MA, USA) in both positive and negative ionization modes. Raw data were converted to mzXML format using ProteoWizard (ver. 3.0), and the XCMS package (ver. 3.1) was used for peak identification, filtering, and alignment [21]. Metabolites were annotated by mapping with public databases, including the standard BioNovoGene database (http://www.bionovogene.com), Metlin (http://metlin.scripps.edu), Mzclound (https://www.mzcloud.org), LipidMaps (http://www.lipidmaps.org), Massbank (http://www.massbank.jp), and the Human Metabolome Database (http://www.hmdb.ca). Multivariate statistical analysis, including partial least squares discriminant analysis (PLS-DA) and orthogonal partial least squares discriminant analysis (OPLS-DA), was performed. Differentially expressed metabolites (DEMs) between groups were screened by the criteria of VIPs > 1 and *p* < 0.05. The DEMs were functionally annotated with the KEGG database.

### 2.8. Integrated Transcriptomic and Metabolomic Analysis

An integrative analysis of the DEGs and DEMs was conducted to identify significantly changed biological pathways. Pearson’s correlation analysis was performed to evaluate the relationships between the DEGs and DEMs. Pairs with a Pearson’s correlation coefficient (|PCC|) > 0.80 and *p* < 0.05 were considered significantly correlated. Furthermore, DEGs and DEMs were matched with the KEGG database to reveal their common pathways.

### 2.9. Validation of Transcriptomic Data

To validate the RNA-seq findings, quantitative real-time PCR (qRT-PCR) was conducted using the same RNA samples prepared for sequencing. Nine DEGs were selected for verification. Each sample was run with three technical replicates. The primers used in qRT-PCR are listed in Table S1. β-actin was selected as the reference gene for normalization. The 2^−△△CT^ method was applied to calculate the fold changes in the mRNA levels in T7 relative to CG [22].

### 2.10. Statistical Analysis

The experimental unit used in this research was the tank. Histological parameters (*n* = 3) were analyzed with the Kruskal–Wallis H test, followed by Dunn’s post hoc test for multiple comparisons. The qRT-PCR validation results and α diversity indices of the intestinal microbiota, including Chao1, Shannon, Good’s coverage, Observed species, Simpson, Faith’s PD, and Pielou’s evenness, were evaluated using the Mann–Whitney U test. All statistical analyses were conducted with GraphPad Prism 9 software (GraphPad Software Inc., La Jolla, CA, USA). Data were expressed as mean ± standard error of the mean (SEM). Statistical significance was defined as *p* < 0.05.

## 3. Results

### 3.1. Histopathological Injury and Intestinal Morphology

**Figure 1:**
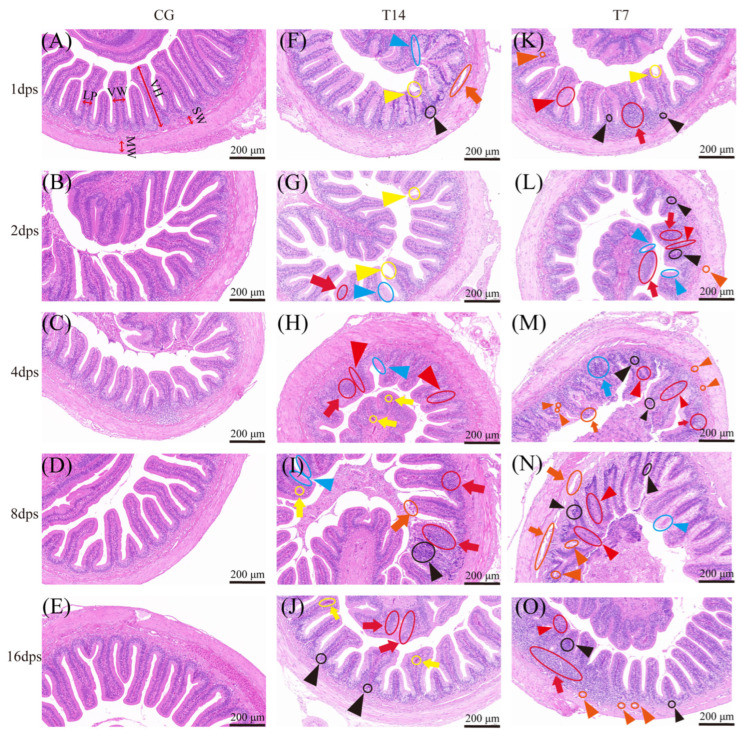
Intestinal histopathology in Chinese soft-shelled turtles observed at sequential time points after acute cold stress. CG (**A**–**E**): 28 °C control group. T14 (**F**–**J**): 14 °C cold stress group. T7 (**K**–**O**): 7 °C cold stress group. VH indicates villus height, VW indicates villus width, LP indicates lamina propria width, SW indicates submucosal width, and MW indicates muscularis width. Histopathological injuries in the intestine include mucosal epithelial degeneration (blue triangle), villus splitting (yellow triangle), villus fusion (red triangle), vacuolation (orange triangle), inflammatory cell infiltration (black triangle), villi fusion (blue thick arrow), necrosis and desquamation of the mucosal epithelium (orange thick arrow), lymphoid hyperplasia (red thick arrow), and hyperemia (yellow thick arrow). Microscope magnification: 200×. Scale bar: 200 μm. “dps” indicates days post cold stress.

The histopathological analysis revealed cold-stress-induced microstructural damage in the intestine (Figure 1). The small intestine in the CG maintained normal structure throughout the experiment, with intact, evenly arranged villi and mucosal epithelium. Mild injuries appeared in both T14 and T7 from 1 to 2 dps (Figure 1F,G,K,L), including inflammatory cell infiltration, lymphoid hyperplasia, mucosal epithelial degeneration, and villus defects such as villus splitting and fusion. Lesions worsened from 4 to 8 dps (Figure 1H,I,M,N), particularly in T7, where severe villus fusion, erosion, extensive epithelial necrosis and desquamation, and obvious fissures in the muscle layer and submucosa were observed.

By 16 dps (Figure 1J,O), histological damage was remarkably ameliorated in both T14 and T7. The villi exhibited well-defined borders, largely intact architecture, and uniform distribution, although mild inflammatory cell infiltration and lymphoid hyperplasia persisted.

**Figure 2:**
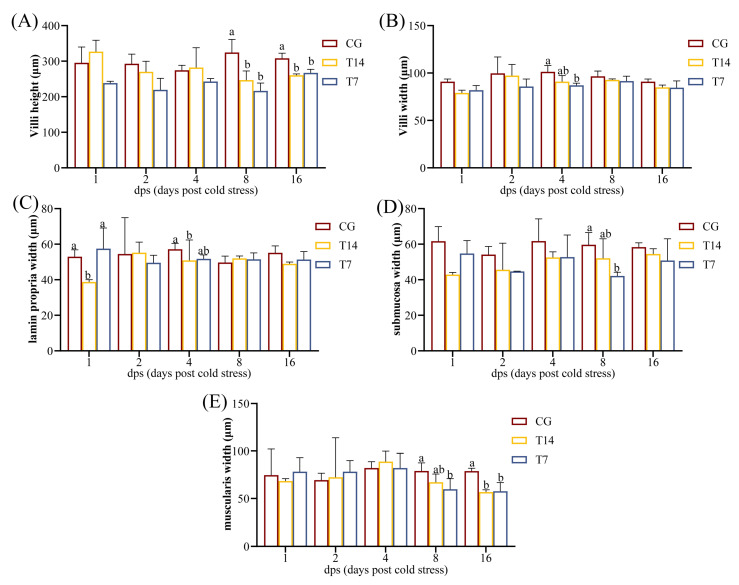
Effect of acute cold stress on the intestinal microstructure of the Chinese soft-shelled turtle at different time points (*n* = 3). (**A**) Measurement of the intestinal villus height. (**B**) Measurement of the intestinal villus width. (**C**) Measurement of the intestinal lamina propria width. (**D**) Measurement of the intestinal submucosal width. (**E**) Measurement of the intestinal muscularis width. Values are expressed as mean ± SEM. Different lowercase letters (a and b) denote statistically significant differences (*p* < 0.05). “CG”: 28 °C control group. “T14”: 14 °C cold stress group. “T7”: 7 °C cold stress group.

To evaluate the effect of acute cold stress on intestinal morphology, villus height, villus width, lamina propria width, submucosal width, and muscularis layer width were measured (Figure 2). Compared with the CG, the villus height (Figure 2A) in the T14 and T7 groups was significantly reduced at 8 and 16 dps (*p* < 0.05). T7 exhibited significantly narrower villus width (Figure 2B) than the CG at 4 dps (*p* < 0.05). The lamina propria width (Figure 2C) was also reduced in the T14 group at 1 and 4 dps (*p* < 0.05). The submucosal width (Figure 2D) in T7 was smaller than in CG at 8 dps (*p* < 0.05). Additionally, the muscularis layer width (Figure 2E) in T14 showed significant shortening at 16 dps (*p* < 0.05), while that of T7 showed significant reductions after both 8 and 16 dps (*p* < 0.05).

Histopathological injuries and morphological changes in the intestine were observed in a time-dependent manner under both 14 °C and 7 °C stress conditions. Notably, the intestinal damage caused by 7 °C low-temperature stress was more severe than that in the 14 °C group. Given the substantial damage caused by 7 °C exposure at 8 dps, small intestinal samples from T7 and CG at 8 dps were selected for comprehensive multi-omics analysis.

### 3.2. Intestinal Microbial Composition

**Figure 3:**
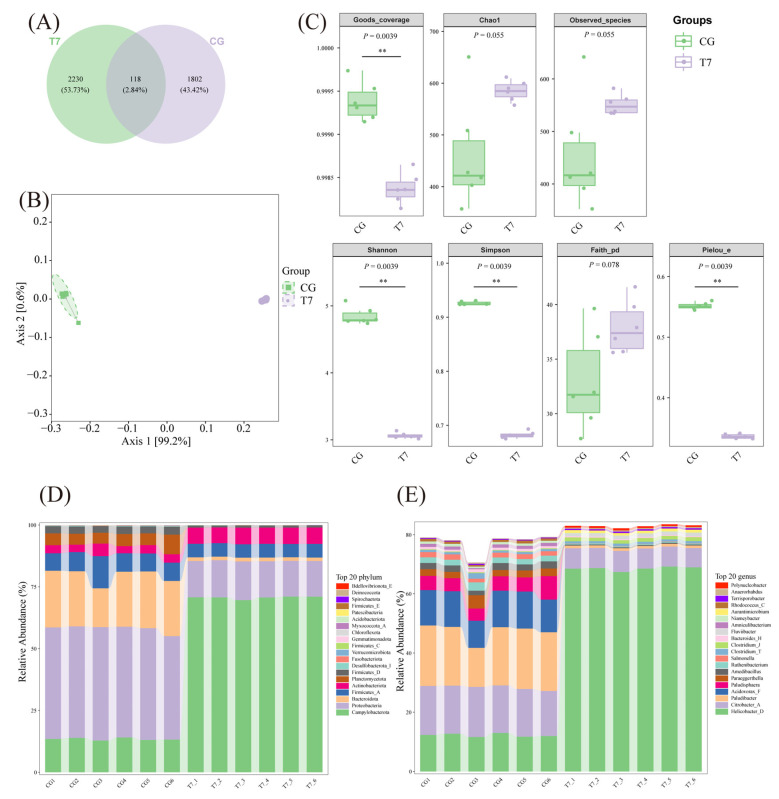
Intestinal microbiome changes in Chinese soft-shelled turtle under cold exposure between CG and T7. (**A**) Venn diagram showing the number of operational taxonomic units (OTUs). (**B**) Alpha diversity of microbiota. (**C**) Principal coordinate analysis (PCoA) of microbial communities. (**D**) Composition of the microbial community at the phylum level. Data in the figure represent the analysis results based on six pooled gut samples from CG and T7 (*n* = 6). Each sample represents a blend of intestines from two turtles within the same tank. (**E**) Composition of the microbial community at the genus level. Data obtained from the same sample as (D). “CG”: 28 °C control group. “T7”: 7 °C cold stress group. ** indicates *p* < 0.01.

A total of 1802 and 2230 OTUs were sequenced in CG and T7, respectively, with 118 OTUs shared between the two groups (Figure 3A). The alpha diversity analysis (Figure 3B) consisted of multiple indices: Good’s coverage (representing sequencing depth), Observed species and Chao1 (indicating species richness), Simpson and Shannon (reflecting species diversity), Faith’s PD (indicating phylogenetic diversity), and Pielou’s evenness. The Good’s coverage values exceeded 99.50% in both groups, demonstrating comprehensive sample coverage. Compared with CG, the Simpson, Shannon, and Pielou’s evenness in T7 showed lower values (*p* < 0.05). The *p* values of Chao1 index (*p* = 0.055), observed species index (*p* = 0.055), and Faith pd index (*p* = 0.078) were close to 0.05. Given the small sample size and the limited statistical power of non-parametric tests employed, these results suggest that these parameters might reach statistical significance in a larger-scale trial. PCoA based on OUT levels revealed clear separation between the two groups, with axes 1 and 2 accounting for 99.2% and 0.6% of the variations, respectively (Figure 3C). The assessment of the microbial community structure found distinct composition at both the phylum (Figure 3D) and genus levels (Figure 3E). The T7 group was dominated by Campylobacterota, Proteobacteria, and Actinobacteriota at the phylum level, and by *Helicobacter* (68.56%) and *Citrobacter* (6.83%) at the genus level. In contrast, the CG was mainly composed of Proteobacteria, Bacteroidota, and Campylobacterota, with *Paludibacter* (18.87%) and *Citrobacter* (16.17%) as the major genera. These results indicate that acute cold stress significantly altered the structure and diversity of the intestinal microbial community.

### 3.3. Alterations in Phenotypic Characteristics of Intestinal Bacteria

**Figure 4:**
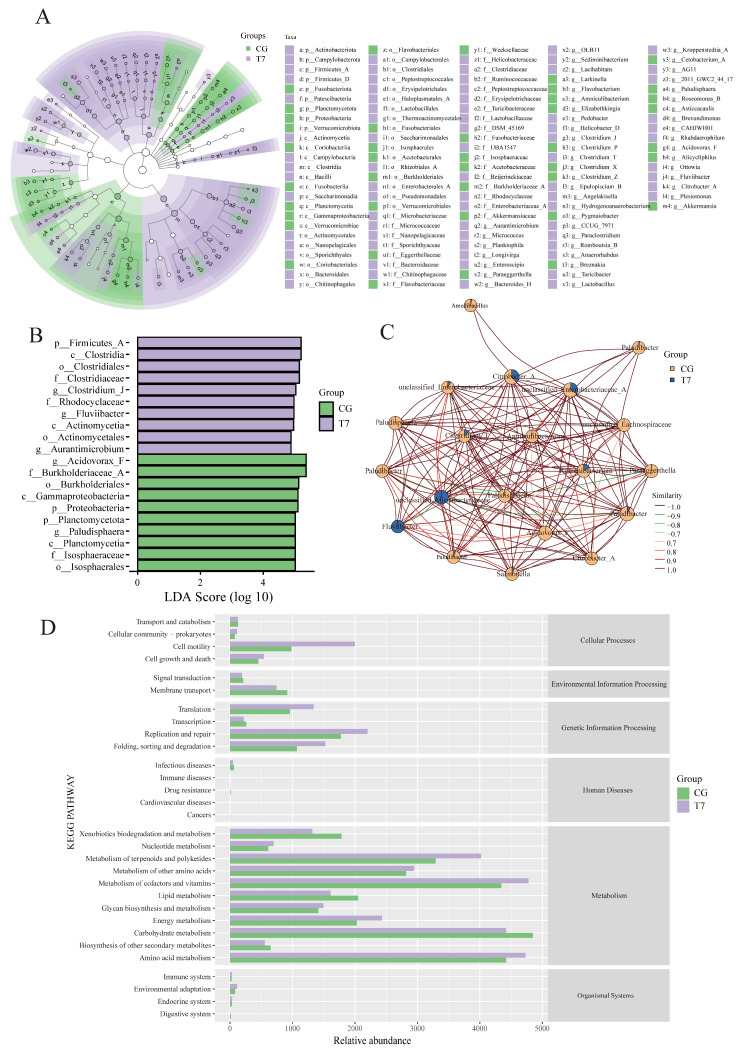
Intestinal indicator species analysis of microbial communities in the Chinese soft-shelled turtle between CG and T7. (**A**) LEfSe multilevel species hierarchy tree. (**B**) Linear discriminant analysis (LDA) score of LEfSe-PICRUSt. (**C**) Bacterial community network of different taxa. (**D**) Functional prediction of intestinal microbiota. “CG”: 28 °C control group. “T7”: 7 °C cold stress group.

To better characterize the microbial composition variations under acute cold stress, cladogram and LEfSe analyses were performed (Figure 4A). T7 showed enrichment of five phyla, five classes, 16 orders, 18 families, and 32 genera, while the CG exhibited the dominance of four phyla, five classes, seven orders, eight families, and 17 genera. The LDA effect size distribution (Figure 4B) further identified the top 10 dominant taxa (LDA score > 4). In T7, the predominant taxa were the genera *Clostridium J*, *Fluviibacter*, and *Aurantimicrobium*, the families Clostridiaceae and Rhodocyclaceae, the orders Clostridiales and Actinomycetales, the classes Clostridia and Actinomycetia, and the phylum Firmicutes A.

The bacterial correlation networks (Figure 4C) revealed distinct network properties between groups: the T7 exhibited fewer nodes, lower density, and reduced clustering coefficients compared to CG, indicating disrupted bacterial connectivity and weakened community stability. Functional prediction showed that acute cold stress significantly altered metabolic pathways (Figure 4D). The pathways “cell motility”, “metabolism of terpenoids and polyketides”, and “folding, sorting and degradation” were enhanced, whereas the “Xenobiotic biodegradation and metabolism”, “lipid metabolism”, and “carbohydrate metabolism” pathways were suppressed. These findings demonstrated that acute cold stress restructured microbial community architecture and reprogrammed metabolic capacity, potentially affecting host energy homeostasis and stress adaptation.

### 3.4. Transcriptomic Profiling of Intestine Under Acute Cold Stress

#### 3.4.1. Sequencing and Functional Enrichment of DEGs

Transcriptomic sequencing revealed approximately 52 million raw reads per group (Table S2). After quality control, over 50 million clean reads were retained in each group, with more than 83% successfully mapped to the reference genome. All samples exhibited high sequencing quality, with Q20 and Q30 values over 96% and GC contents averaging approximately 48%. These data confirmed the RNA-seq data were reliable for subsequent analysis.

**Figure 5:**
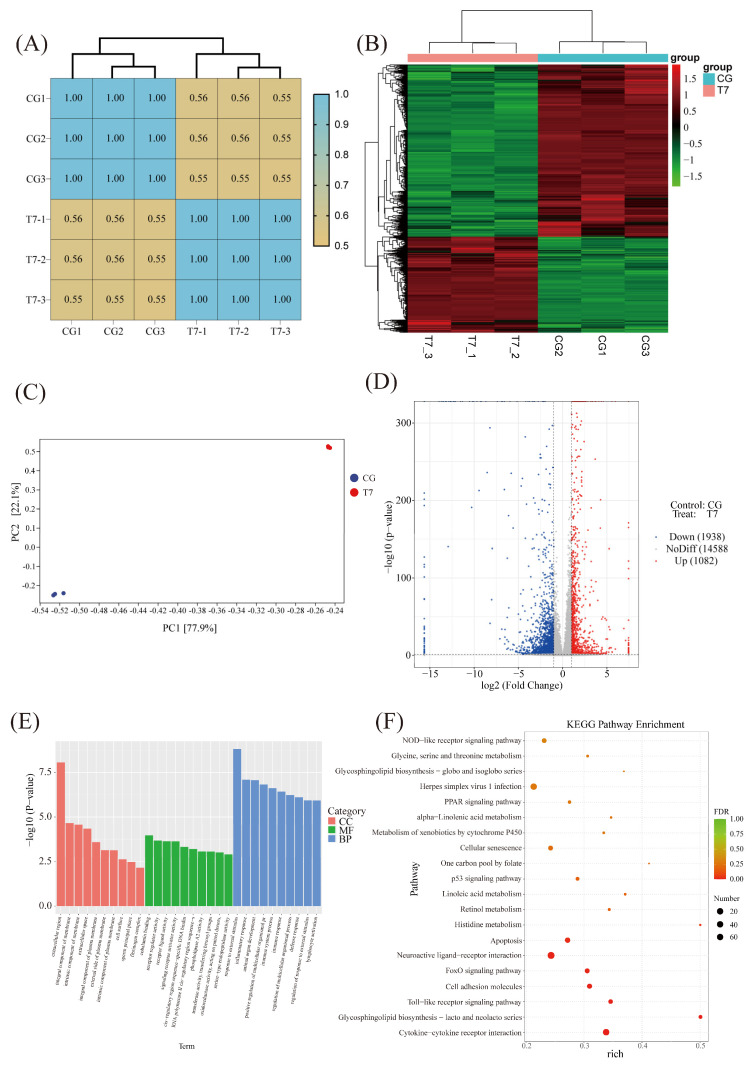
Comparative transcriptomic analysis in intestines of Chinese soft-shelled turtle between CG and T7. (**A**) Correlation heatmap of all samples. (**B**) Heatmap of all expressed gene profiles. (**C**) Principal component analysis (PCA) based on all expressed genes. (**D**) Volcano plot showing the differentially expressed genes (DEGs) in the intestine of Chinese soft-shelled turtle. (**E**) Gene Ontology (GO) enrichment analysis of the DEGs. The top significantly enriched GO terms are displayed: biological process (BP), molecular function (MF), and cellular component (CC). (**F**) KEGG analysis of the DEGs. “CG”: 28 °C control group.

Three biological repeats with PCCs over 0.95 in all groups showed high reproducibility (Figure 5A). Both clustering (Figure 5B) and principal component analysis (PCA) (Figure 5C) revealed obvious distinctions between CG and T7. The volcano plot exhibited 3020 DEGs, including 1082 upregulated and 1938 downregulated genes (Figure 5D). GO analysis uncovered significantly enriched terms in biological process (BP), molecular function (MF), and cellular component (CC) (Figure 5E). The key DEGs in BP included “response to external stimulus” (Go:0009605) and “inflammatory response” (Go:0006954). Enriched terms in CC were involved in “extracellular region” (Go:0005576) and “integral component of membrane” (Go:0016021), while MF was dominated by “cobalamin binding” (Go:0031419) and “receptor regulator activity” (Go:0030545).

The KEGG enrichment analysis revealed 20 significantly enriched pathways (Figure 5F), 10 of which were involved in immune function (Table S3), such as “cytokine–cytokine receptor interaction” (including *Edar*, *Cntf*, *Il17b*, *Epor*, *Cxcr5*, *Ccr6*, *Bmpr1b*, *and Ccr7*), “FoxO signaling pathway” (including *Cdkn1a*, *Igf1*, *Bcl6*, *Bnip3*, *Tnfsf10*, *S1pr4*, *Egf*, and *Plk2*), and “apoptosis” (including *Tuba1c*, *Ctsz*, *Ctsb*, *Kras*, *Hras*, *Pik3ca*, *Bcl2l11*, *Gadd45a*, *Pmaip1*, *Ddit3*, and *Fos*).

#### 3.4.2. qRT-PCR Verification

The reliability of the transcriptomic data was validated by quantifying the mRNA expression levels of nine randomly selected genes using qRT-PCR. (Figure S2). The qRT-PCR results showed consistent expression patterns with transcriptomic data, confirming the accuracy and reproducibility of RNA-seq analysis.

### 3.5. Metabolomic Profiling of Intestine

#### 3.5.1. Multivariate Statistical Analysis

**Figure 6:**
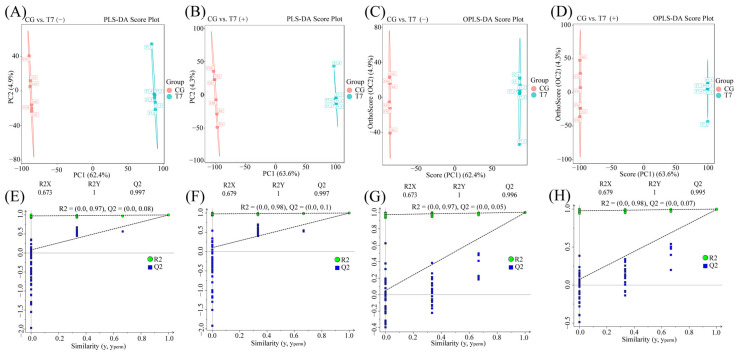
Multivariate statistical analysis of metabolites in the Chinese soft-shelled turtle between CG and T7. The differential expressed metabolites (DEMs) were analyzed using Partial Least Squares Discriminant Analysis (PLS-DA) in negative (**A**) and positive (**B**) ion modes. Orthogonal Projections to Latent Structures Discriminant Analysis (OPLS-DA) was subsequently applied to the DEMs in negative (**C**) and positive (**D**) ion modes. Permutation tests were conducted to validate the PLS-DA models (**E**,**F**) and the OPLS-DA models (**G**,**H**). Permutation test of OPLS-DA models in negative ion mode (**G**) and positive ion mode (**H**). “R2Y” indicates the explanatory rate. “Q2Y” indicates the predictive ability. “CG”: 28 °C control group. “T7”: 7 °C cold stress group.

Multivariate statistical models effectively distinguished the metabolic profiles between CG and T7. Both PLS-DA (Figure 6A,B,E,F) and OPLS-DA models (Figure 6C,D,G,H) demonstrated clear separation in POS and NEG. The contribution rate and predictability of two models respectively evaluated by R2Y and Q2 parameters indicated their reliability and separating capacity for group discrimination, with R2Y = 1 cum and Q^2^ > 0.995 cum.

#### 3.5.2. Screening for Differential Metabolites

**Figure 7:**
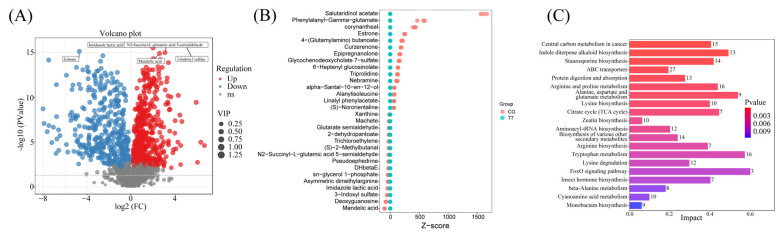
Comparative metabolomic analysis in intestines of Chinese soft-shelled turtle between CG and T7. (**A**) Volcano plot of differentially expressed metabolites (DEMs). (**B**) Z-score plot showing the top 30 DEMs. (**C**) KEGG analysis of DEMs. “CG”: 28 °C control group. “T7”: 7 °C cold stress group.

A comparative metabolomic analysis identified 886 DEMs between CG and T7, with 495 upregulated and 391 downregulated (Figure 7A). Z-score plots (Figure 7B) highlighted the most significantly decreased metabolites of salutaridinol acetate, phenylalanyl-γ-glutamate, corynantheal, estrone, and 4-(glutamylamino) butanoate, as well as elevated mandelic acid, deoxyguanosine, 3-indoxyl sulfate, imidazole lactic acid, and asymmetric dimethylarginine in T7. The KEGG analysis revealed predominantly enriched pathways (Figure 7C), including “indole diterpene alkaloid biosynthesis”, “ABC transporters”, “arginine and proline metabolism”, “lysine biosynthesis”, and “citrate cycle”.

### 3.6. Integrated Analysis of Transcriptome and Metabolome

**Figure 8:**
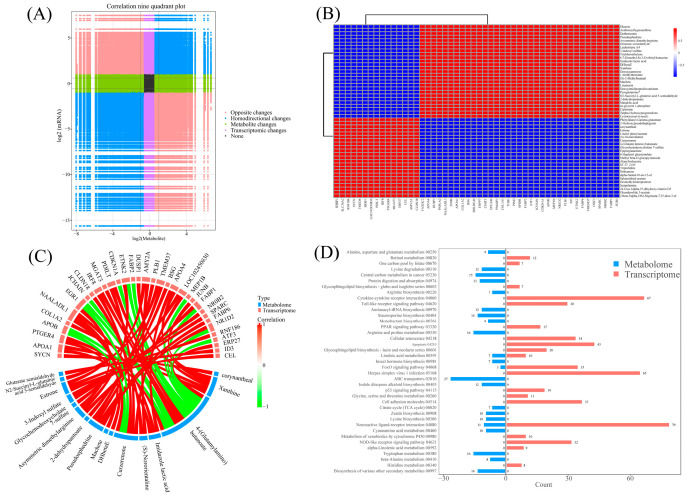
Integrated analysis of metabolome and transcriptome in intestine of Chinese soft-shelled turtle between CG and T7. (**A**) Nine-quadrant diagram illustrating the correlation between differentially expressed genes (DEGs) and differentially expressed metabolites (DEMs). (**B**) Heatmap of the correlation between DEGs and DEMs. (**C**) Chord diagram exhibiting significant association of DEGs and DEMs. (**D**) KEGG pathway enriched by both DEGs and DEMs. “CG”: 28 °C control group. “T7”: 7 °C cold stress group.

Pearson correlation analysis identified significant relationships between DEGs and DEMs (Figure 8A,B), with 50 gene–metabolite pairs showing strong correlations (|PCC| > 0.80 and *p* < 0.05).

**Figure 9:**
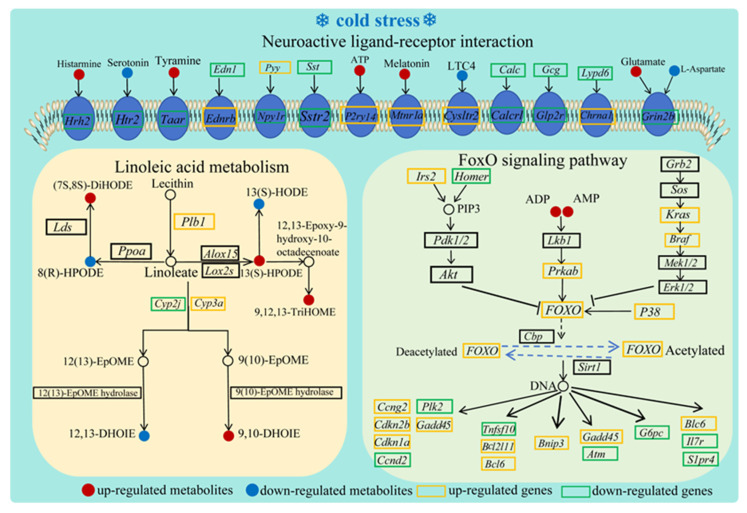
Network maps illustrating the predominant genes, metabolites, and signaling pathways responding to cold stress.

Genes such as *faxdc2*, *apoa4*, *dusp1*, *pnpla2*, and *naaladl1* exhibited positive correlations with metabolites, such as dhurrin, arabinosylhypoxanthine, dothistromin, pseudoephedrine, and asymmetric dimethylarginine, but negative correlations with phenylalanyl-gamma-glutamate, 2-hydroxypseudobaptigenin, corynantheal, estrone, and linalyl phenylacetate. Conversely, reciprocal patterns were found for *rspr1*, *slc34a2*, *rnf186*, *sycn*, and *tmed6* expression. A chord diagram (Figure 8C) further delineated specific correlations between top-ranked DEGs and DEMs. For instance, 4-(Glutamylamino)-butanoate was positively associated with *Pdilt* and *Mgat3* but negatively related to *Apob* and *Egr1*. Three critical pathways—“linoleic acid metabolism”, “FoxO signaling pathway”, and “neuroactive ligand–receptor interaction”—were enriched in both DEGs and DEMs, suggesting their pivotal role in acute cold stress response (Figure 8D and Figure 9).

## 4. Discussion

Exposure to low temperature triggers significant physiological, immunological, and metabolic dysregulation in aquatic species, often leading to observable morphological alterations and tissue damage of the intestine [23]. While the detrimental effects of extreme temperatures on intestinal health are known in several aquatic species [15,24,25], the intestinal response of Chinese soft-shelled turtles to acute cold stress remains poorly characterized. Consequently, the histology, microbiome, transcriptome, and metabolome analyses were integrated to elucidate the adaptive mechanisms of the intestine under acute cold exposure.

### 4.1. Effects of Low Temperature on Intestinal Structure

Histopathological injury and intestinal morphology are key indicators for evaluating intestinal health under environmental stress [26,27]. Under normal conditions, the intestinal epithelium of *Pelodiscus sinensis* is characterized by a pseudostratified columnar structure with tightly connected enterocytes and abundant mucous cells [28]. In this study, exposure to 14 °C and 7 °C induced progressive intestinal damage in a temperature- and time-dependent manner. Early stages (from 1 to 2 dps) featured inflammatory cell infiltration and lymphoid hyperplasia, implying the onset of inflammatory lesions [29]. By 4–8 dps, severe villus detachment, necrosis, and submucosal and muscular layer damage were observed. A notable amelioration of intestinal morphology was observed at 16 dps. As a species capable of winter dormancy, Chinese soft-shelled turtles appear to activate intrinsic adaptive mechanisms to restore homeostasis, such as regulating stress-protective genes and mobilizing cellular repair systems [5]. Previous studies on hibernation in this species show that the intestinal villi remain structurally intact during dormancy, with the up-regulation of intestinal barrier proteins (ZO-1 and Cx43) and innate immune genes (*Tlr2* and *Tlr4*) [30]. We deduced that similar adaptive mechanisms possibly contributed to the histological repair at 16 dps observed in our study. Generally, these results demonstrated the intestinal capacity to restore homeostasis following initial cold-induced damage.

Quantitative morphological analysis demonstrated significant reductions in several intestinal parameters during cold stress, including villus height and width, as well as submucosal width and muscularis layer width. These morphological alterations likely impaired intestinal barrier function, nutrient absorption, and immune defense, consistent with previous observations in yellow catfish [31] and zebrafish [24]. Notably, 7 °C treatment elicited more severe damage than 14 °C, suggesting a temperature-dependent nature of acute cold stress injury.

### 4.2. Effects of Low Temperature on the Intestinal Microbiome

The intestinal microbiome plays a pivotal role in maintaining intestinal functions, including digestion, nutrient absorption, and immune regulation. In aquatic species, temperature is a key environmental factor shaping microbial composition [32]. In the current research, α-diversity indices (Simpson, Shannon, and Pielou’s evenness) were significantly reduced, indicating decreased microbial diversity and evenness. Further analysis at the phylum level found that cold stress reduced the abundance of Proteobacteria and Bacteroidota—typical dominant phyla in healthy turtles—but increased Actinobacteriota. This result is similar to previous reports in teleosts [15] and the same turtle [33], implying that acute cold stress profoundly influenced the gut microbiota architecture of the Chinese soft-shelled turtle. At the genus level, potentially pathogenic taxa such as *Helicobacter* [34] and *Citrobacter* [35] were enriched in the 7 °C group, implying elevated disease risk under acute cold stress. The enrichment of *Helicobacter* and *Citrobacter* coincided with severe intestinal damage and metabolic disturbances, suggesting a possible pathological association that needs further validation. Functional analyses showed that microbiota lipid metabolism and carbohydrate metabolism were suppressed at 7 °C, while the energy and amino acid metabolism pathways were elevated, indicating a metabolic shift toward supporting host energy demands.

The relationship between host physiology and gut microbiota under cold stress might be bidirectional. On one hand, intestinal damage induced by acute cold stress, such as epithelial necrosis and inflammation, can alter the gut microenvironment, which promotes the growth of opportunistic pathogens and impairs host metabolic and immune function. This is consistent with seasonal studies of Chinese soft-shelled turtles, where winter restructuring of the gut microbiota enriches conditional pathogens such as *Aeromonas* and *Edwardsiella*. These alterations potentially contribute to post-hibernation disease susceptibility [36]. On the other hand, microbial changes may also reflect adaptation to cold stress. Intestinal villus atrophy under low temperatures reduces nutrient absorption capacity. Meanwhile, the microorganisms involved in “energy metabolism” and “amino acid metabolism” increased, suggesting that the microbiota may reallocate metabolic resources to restore host energy homeostasis. Similar adaptive metabolic alterations have been reported in cold-stressed *Micropterus salmoides* [15].

### 4.3. Functional Gene and Metabolite Screening Under Acute Cold Stress

Aquatic animals can modulate their immune systems in response to temperature fluctuations. As the first line of defense against pathogens [37], the innate immune system utilizes pattern recognition receptors (PRRs) to identify molecular signatures associated with pathogens or cellular damage, thereby triggering downstream immune responses [38]. Our findings demonstrated the significant down-regulation of multiple Toll-like receptor signaling pathway genes (*Tlr2*, *Tlr4*, *Tlr5*, *Tlr7,* and *Tlr8*) and NOD-like receptor signaling pathway genes (*Traf6*, *Traf2*, *Casr*, *Rnasel*, *Pstpip1*, *Plcb2*, *Atg5,* and *Mfn2*) in the 7 °C group, indicating that the pathogen recognition ability may be impaired. These results aligned with previous reports in tilapia, where genes encoding TLRs and NLRs are suppressed after 10 °C exposure [39]. In addition, genes involved in apoptosis (*Tuba1c*, *Ctsz*, *Ctsb*, *Kras*, *Hras*, *Pik3ca*, *Bcl2l11*, *Gadd45a*, *Pmaip1*, *Ddit3*, and *Fos*) and the p53 signaling pathway (*Serpine1*, *Sesn2*, *Ccng2*, *Igf1*, *Mdm2*, *Gadd45a*, *Pmaip1*, and *Cdkn1a*) were significantly upregulated in this research, indicating that cold stress induces intestinal apoptosis, corroborating the aforementioned histopathological damage.

Metabolomic analysis further revealed disturbances in lysine metabolism, with altered levels of key intermediates such as oxoglutaric acid, L-lysine, and L-aspartic acid. As a nutritionally important amino acid, lysine plays an important role in growth regulation and immune function in aquatic species [40]. Lysine supplementation is able to enhance organismal resilience to high-temperature stress [41]. Our study results indicate that lysine supplementation has the potential to mitigate acute cold-stress-induced metabolic disturbances. In addition, multiple intermediates of the TCA cycle showed significant changes, such as oxoglutaric acid, oxaloacetate, succinic acid, fumaric acid, DL-malic acid, citric acid (CA), and aconitate. CA is known to enhance gut health and alleviate oxidative stress in aquatic species [42,43,44]. Its decrease in our results indicates that restoring TCA cycle homeostasis through CA supplementation could be a potential strategy to mitigate intestinal damage under cold stress.

### 4.4. Crucial Pathways Responding to Low-Temperature Stress

By integrating DEG and DEM data, we identified three key pathways that mediate the intestinal response of the Chinese soft-shelled turtle to acute cold stress, including “linoleic acid metabolism”, “neuroactive ligand–receptor interaction”, and the “FoxO signaling pathway”.

In response to cold stress, poikilothermic vertebrates utilize several key strategies: modulating cell membrane fluidity, producing antifreeze proteins, and increasing the synthesis of molecular chaperones [45]. Linoleic acid, a key component of membrane phospholipids, contributes to maintaining membrane fluidity. This study revealed significant alterations in genes (*Plb1*, *Cyp2j*, and *Cyp3a*) and metabolites ((12Z)-9,10-Dihydroxyoctadec-12-enoic acid (9,10-DHOME), 13S-Hydroperoxy-9Z,11E-octadecadienoic acid (13(S)-HPODE), (12Z)-9,10-Dihydroxyoctadec-12-enoic acid (9,10-DHOME), (13S)-Hydroxyoctadecadienoic acid (13(S)-HODE), and 9(S),12(S),13(S)-Trihydroxy-10(E)-octadecenoic acid (9,12,13-TriHOME)) related to linoleic acid metabolism. *Plb1* activation leads to the down-regulation of *Cyp2j* expression and the up-regulation of *Cyp3a* expression, resulting in a decrease in 12,13-DHOME and an increase in 9,10-DHOME. On the other hand, *Plb1* can specifically catalyze the conversion of 13(S)-HPODE to 9,12,13-TriHOME, rather than 13(S)-HODE. This result is similar to previous reports on *Hemiphyllodactylus yunnanensis* [46] and *Eremias argus* [47], suggesting that the activation of linoleic acid metabolism to maintain cell membrane fluidity may represent a universal physiological mechanism in poikilothermic vertebrates for coping with cold stress.

Organisms eliminate injured cells via apoptotic processes to maintain normal physiological function [48]. The FoxO signaling pathway is a critical regulator of apoptosis [49]. In *Brachymystax lenok tsinlingensis*, FoxO suppresses apoptosis through the up-regulation of the anti-apoptotic gene Bcl-6. Similar regulatory patterns have been reported in cold-challenged carps [50] and tsinling lenok trout [51]. Our study observed increased mRNA levels of pro-apoptotic genes (*Foxo4*, *Prkab*, *Bcl2l11*, *Bcl6*, *Bnip3*, and *Cdkn1a*) and metabolites such as adenosine diphosphate (ADP) and adenosine monophosphate (AMP). ADP and AMP, as key intracellular energy-sensing molecules, regulate the expression of *Prkab*. The activation of *Prkab* regulates the expression of the *Foxo4* gene, further initiating the transcription of pro-apoptotic genes such as *Bcl6*, *Bnip3*, and *Bcl2l11*. This pathway was also identified in the spleen of Chinese soft-shelled turtles under similar conditions [52], highlighting its systemic role in stress adaptation. Neuroactive ligand–receptor (NLR) interaction, essential for intercellular communication and stress signal transduction, significantly changed. Similarly, NLR activation has been documented in the brains of yellow drum [53], *Coilia nasus* [54], and gynogenetic mrigal carp [55] under cold stress. Additionally, intestinal NLR regulation has been observed in yellow pond turtles [56]. Key genes (*Adcyap1*, *Adora2a*, *Adra2a*, *Agtr1*, and *Agtr2*) and metabolites (serotonin, L-glutamic acid, L-aspartic acid, melatonin, and taurine) were remarkably altered, suggesting that NLR signaling might regulate neuronal activity to respond to acute cold stress in Chinese soft-shelled turtles.

In general, multi-omics data indicate that cold stress leads to the disruption of intestinal microbiota homeostasis, enrichment of potentially pathogenic bacteria, and suppression of microbial metabolic functions, particularly in lipid and carbohydrate metabolism. This dysbiosis, together with direct cold stress, triggers host metabolic reprogramming, characterized by altered TCA cycle intermediates, activated linoleic acid metabolism, and modified neuroactive ligand–receptor interactions. Concurrently, cold stress activates apoptosis and p53 pathways to clear and repair damaged epithelium, while suppressing immune recognition via TLR/NLR signaling. These coordinated responses ultimately lead to intestinal morphological damage, metabolic dysfunction, and weakened immunity.

### 4.5. Impact of Acute Cold Stress on Systemic Metabolism via the Gut–Liver Axis

Cold stress not only disrupts intestinal flora imbalance, but also synergistically disrupts intestinal and hepatic metabolism and function through the gut–liver axis. The intestinal microbiota and liver communicate bidirectionally via metabolites, immune signals, and barrier integrity, playing a pivotal role in low-temperature adaptation [57]. Spearman correlation analysis (Figure S3) between liver metabolites from our published paper [18] and intestinal microbiota revealed correlations between specific gut bacterial taxa and hepatic metabolites, suggesting the microbiota-mediated regulation of liver function.

Notably, *Helicobacter* abundance correlated strongly with uric acid, a biomarker and pathogenic driver of non-alcoholic fatty liver disease (NAFLD). It can promote reactive oxygen species (ROS) generation by NADPH oxidase activation, inducing oxidative stress and activating the NF-κB signaling pathway to upregulate inflammatory factors [58]. Similar mechanisms have also been reported in gut–liver axis injury models [59]. *Citrobacter* enrichment was correlated with elevated hepatic pro-inflammatory metabolites such as hypoxanthine and lysophosphatidic acid (LysoPA). Hypoxanthine accumulation reflects purine metabolic dysregulation and exacerbates hepatocyte oxidative damage by enhancing ROS production [60]. LysoPA, at high local concentrations, disrupts cell membranes and induces cytotoxicity [61]. These microbiota–metabolite–liver regulatory patterns align with observations in murine models [59,62]. Consistent with our previous histopathological damage in the liver, these findings suggest that specific bacteria may aggravate intestinal barrier damage and hepatic inflammation by releasing endotoxins or pro-inflammatory factors.

## 5. Conclusions

This study comprehensively investigated the effects of acute cold stress on intestinal health in Chinese soft-shelled turtles. Exposure to 14 °C and 7 °C induced significant intestinal damage, with more severe pathology at 7 °C. Cold stress also disrupted the gut microbiota and altered key genes and metabolites. Enriched pathways included “linoleic acid metabolism”, “neuroactive ligand–receptor interaction”, and the “FoxO signaling pathway”. Furthermore, cold-induced dysbiosis, marked by the enrichment of *Helicobacter* and *Citrobacter*, might exacerbate systemic injury via metabolite-mediated gut–liver axis crosstalk, highlighting this axis as central to the host’s cold stress response.

The findings of this study offer promising insights, yet certain limitations should be considered. These findings are preliminary due to limited omics sample sizes (transcriptomics *n* = 3, metabolomics *n* = 6 per group) and controlled laboratory conditions. Future studies should validate mechanisms in larger, field-relevant cohorts and address sex-specific adaptations in mature turtles. The inferred functional data from omics tools also require experimental confirmation. In addition, our findings reflect responses from a mixed-sex juvenile population without considering potential sex-specific adaptations. Future studies should investigate sex-dependent adaptive mechanisms to cold stress in Chinese soft-shelled turtles.

Our work provides a basis for three potential future applications: (1) developing feed additives from identified metabolites (citric acid and succinic acid) to alleviate cold stress; (2) using key differentially expressed genes in marker-assisted selection for cold-tolerant strains; and (3) targeting the gut–liver axis through microbiota modulation or metabolite supplementation to mitigate systemic cold injury in aquaculture.

## Data Availability

All data generated and analyzed during this study are included in this published article. All raw RNA sequencing data have been submitted in the NCBI Sequence Read Archive (SRA) with the BioProject ID PRJNA1287258.

## Supplementary Materials

The following supporting information can be downloaded at: https://www.mdpi.com/article/10.3390/ani16020256/s1, Figure S1: Sketch of the Intestine division; Figure S2: Pearson correlation analysis of mRNA expression levels of random 9 genes between RNA-seq and real-time PCR results in CG and T7; Figure S3: Spearman correlation analysis between the top 20 intestinal microbiota by abundance and liver metabolites; Table S1: The qRT-PCR primer sequences of the Chinese soft-shelled turtle; Table S2: Overview of the transcriptomic sequencing quality in the CG and T7; Table S3: The DEGs enriched in immune pathways of the intestines in CG and T7.

## References

[1] Liang Y.X., Tian P., Lu Y.K., Qin Q., Wang Z.A., Xiong G., Wang X.Q., Hu Y.Z. (2024). Establishment and population genetic analysis of SNP fingerprinting of Chinese soft-shelled turtle (*Pelodiscus sinensis*). Aquac. Rep..

[2] Wang D., Gao H.Q. (2025). China Fishery Statistical Yearbook.

[3] Zhang Z., Chen B., Yuan L., Niu C. (2015). Acute cold stress improved the transcription of pro-inflammatory cytokines of Chinese soft-shelled turtle against *Aeromonas hydrophila*. Dev. Comp. Immunol..

[4] Pirhalla D.E., Sheridan S.C., Ransibrahmanakul V., Lee C.C. (2015). Assessing Cold-Snap and Mortality Events in South Florida Coastal Ecosystems: Development of a Biological Cold Stress Index Using Satellite SST and Weather Pattern Forcing. Estuaries Coasts.

[5] Reid C.H., Patrick P.H., Rytwinski T., Taylor J.J., Willmore W.G., Reesor B., Cooke S.J. (2022). An updated review of cold shock and cold stress in fish. J. Fish Biol..

[6] Scharsack J.P., Franke F. (2022). Temperature effects on teleost immunity in the light of climate change. J. Fish Biol..

[7] Sun Z.Z., Tan X.H., Liu Q.Y., Ye H.Q., Zou C.Y., Xu M.L., Zhang Y.F., Ye C.X. (2019). Physiological, immune responses and liver lipid metabolism of orange-spotted grouper (*Epinephelus coioides*) under cold stress. Aquaculture.

[8] Chu P., Wang S., Yu W., Wang A., Zong Y., Yin S., Zhao C., Wang T. (2024). The impact of extremely low-temperature changes on fish: A case study on migratory bony fishes (*Takifugu fasciatus*). Aquaculture.

[9] Liu T.Y., Li L., Yang Y.C., Li J.R., Yang X.T., Li L., Zheng Z.Y., Yang B.Y., Zhang P.Y., Liu H.Y. (2025). Effects of chronic cold stress and thermal stress on growth performance, hepatic apoptosis, oxidative stress, immune response and gut microbiota of juvenile hybrid sturgeon (*Acipenser baerii* ♀ × *A. schrenkii* ♂). Fish Shellfish Immunol..

[10] Zhang W.Y., Niu C.J., Chen B.J., Storey K.B. (2018). Digital Gene Expression Profiling reveals transcriptional responses to acute cold stress in Chinese soft-shelled turtle *Pelodiscus sinensis* juveniles. Cryobiology.

[11] Lu D.L., Ma Q., Sun S.X., Zhang H., Chen L.Q., Zhang M.L., Du Z.Y. (2019). Reduced oxidative stress increases acute cold stress tolerance in zebrafish. Comp. Biochem. Physiol. Part A Mol. Integr. Physiol..

[12] Zhang S.J., Gong R.X., Zhao N., Zhang Y., Xing L., Liu X.T., Bao J., Li J.H. (2023). Effect of intermittent mild cold stimulation on intestinal immune function and the anti-stress ability of broilers. Poult. Sci..

[13] Quan J.Q., Kang Y.J., Luo Z.C., Zhao G.Y., Ma F., Li L.L., Liu Z. (2020). Identification and characterization of long noncoding RNAs provide insight into the regulation of gene expression in response to heat stress in rainbow trout (*Oncorhynchus mykiss*). Comp. Biochem. Physiol. Part D Genom. Proteom..

[14] Fang M., Lei Z., Ruilin M., Jing W., Leqiang D. (2023). High temperature stress induced oxidative stress, gut inflammation and disordered metabolome and microbiome in tsinling lenok trout. Ecotoxicol. Environ. Saf..

[15] Ma S.B., Lv Y.B., Hou L., Jia Z.M., Lin S., Wang S.D., He X.G., Hou J. (2025). Effect of acute temperature stress on energy metabolism, immune performance and gut microbiome of largemouth bass (*Micropterus salmoides*). Aquac. Fish..

[16] Feng Q.Q., Chen W.D., Wang Y.D. (2018). Gut Microbiota: An Integral Moderator in Health and Disease. Front. Microbiol..

[17] Sepulveda J., Moeller A.H. (2020). The Effects of Temperature on Animal Gut Microbiomes. Front. Microbiol..

[18] Ji L.Q., Shi Q., Chen C., Liu X.L., Zhu J.X., Hong X.Y., Wei C.Q., Zhu X.P., Li W. (2025). Biochemical, Histological, and Multi-Omics Analyses Reveal the Molecular and Metabolic Mechanisms of Cold Stress Response in the Chinese Soft-Shelled Turtle (*Pelodiscus sinensis*). Biology.

[19] Zhang Z.B., Song R.X., Xing X.X., Wang L.W., Niu C.J. (2018). Division of Chinese soft-shelled turtle intestine with molecular markers is slightly different from the morphological and his-tological observation. Integr. Zool..

[20] Wang W.Z., Huang J.S., Zhang J.D., Wang Z.L., Li H.J., Amenyogbe E., Chen G. (2021). Effects of hypoxia stress on the intestinal microflora of juvenile of cobia (*Rachycentron canadum*). Aquaculture.

[21] Smith C.A., Want E.J., O’Maille G., Abagyan R., Siuzdak G. (2006). XCMS: Processing mass spectrometry data for metabolite profiling using nonlinear peak alignment, matching, and identification. Anal. Chem..

[22] Houghton S.G., Cockerill F.R. (2006). Real-time PCR: Overview and applications. Surgery.

[23] Rostagno M.H. (2020). Effects of heat stress on the gut health of poultry. J. Anim. Sci..

[24] Chu P., Li Y.F., Han X.M., Li X.J., Liu Y.X., Tang Z.X., Yin S.W., Wang T. (2025). Mechanism of low temperature-induced intestinal damage in *Danio rerio* and the mitigating effect of alanylglutamine. Water Biol. Secur..

[25] Li R.X., Amenyogbe E., Lu Y., Jin J.H., Xie R.T., Huang J.S. (2023). Effects of low-temperature stress on intestinal structure, enzyme activities and metabolomic analysis of juvenile golden pompano (*Trachinotus ovatus*). Front. Mar. Sci..

[26] Zhang Y.M., Xu W.B., Cheng Y.X., Chen D.Y., Lin C.Y., Li B.Z., Dong W.R., Shu M.A. (2022). Effects of air exposure stress on crustaceans: Histopathological changes, antioxidant and immunity of the red swamp crayfish *Procambarus clarkii*. Dev. Comp. Immunol..

[27] Wiersema M.L., Koester L.R., Schmitz-Esser S., Koltes D.A. (2021). Comparison of intestinal permeability, morphology, and ileal microbial communities of commercial hens housed in conventional cages and cage-free housing systems. Poult. Sci..

[28] Bao H.J., Chen Q.S., Su Z.H., Qin J.H., Xu C.S., Arencibia A., Rodríguez-Ponce E., Jaber J.R. (2017). The study of microanatomy of intestinal epithelium in the Chinese soft-shelled turtle (*Pelodiscus sinensis*). Iran. J. Vet. Res..

[29] Yu Y.M., Sitaraman S., Gewirtz A.T. (2004). Intestinal epithelial cell regulation of mucosal inflammation. Immunol. Res..

[30] Shi Y.H., Vistro W.A., Bai X.B., Wu R.Z., Chen C., Huang Y.F., Fazlani S.A., Tarique I., Yang P., Chen Q.S. (2020). Effect of seasonal variance on intestinal epithelial barriers and the associated innate immune response of the small intestine of the Chinese soft-shelled turtles. Fish Shellfish Immunol..

[31] Hu J.R., Wang L., Wang G.X., Zhao H.X., Lu H.J., Peng K., Huang W., Liu Z.X., Liu D., Sun Y.P. (2024). Selenium Protects Yellow Catfish (*Tachysurus fulvidraco*) from Low-Temperature Damage via the Perspective Analysis of Metabolomics and Intestinal Microbes. Fishes.

[32] Singh B.K., Thakur K., Kumari H., Mahajan D., Sharma D., Sharma A.K., Kumar S., Singh B., Pankaj P.P., Kumar R. (2025). A review on comparative analysis of marine and freshwater fish gut microbiomes: Insights into environmental impact on gut microbiota. FEMS Microbiol. Ecol..

[33] Ji L.Q., Shangguan Y.S., Chen C., Wei C.Q., Zhu J.X., Hong X.Y., Liu X.L., Zhu X.P., Li W. (2025). Dietary Tannic Acid Promotes Growth Performance and Resistance Against *Aeromonas hydrophila* Infection by Improving the Antioxidative Capacity and Intestinal Health in the Chinese Soft-Shelled Turtle (*Pelodiscus sinensis*). Antioxidants.

[34] Hand T.W., Overacre-Delgoffe A.E. (2022). The complex immunological role of *Helicobacter* in modulating cancer. Trends Immunol..

[35] Jabeen I., Islam S., Hassan A.K.M.I., Tasnim Z., Shuvo S.R. (2023). A brief insight into *Citrobacter* species—A growing threat to public health. Front. Antibiot..

[36] Zhang Z.B., Wang D.Q., Xiao P., Liu N., Dalmo R.A., Niu C.J. (2025). Seasonal dynamics of gut microbiota in Chinese soft-shelled turtles (*Pelodiscus sinensis*): Implications for sustainable aquaculture practices. Aquaculture.

[37] Ji L.Q., Chen C., Zhu J.X., Hong X.Y., Liu X.L., Wei C.Q., Zhu X.P., Li W. (2024). Integrated time-series biochemical, transcriptomic, and metabolomic analyses reveal key metabolites and signaling pathways in the liver of the Chinese soft-shelled turtle (*Pelodiscus sinensis*) against *Aeromonas hydrophila* infection. Front. Immunol..

[38] Franchi L., Muñoz-Planillo R., Núñez G. (2012). Sensing and reacting to microbes through the inflammasomes. Nat. Immunol..

[39] Huang D.Y., Liang H.L., Zhu J., Ren M.C., Ge X.P. (2022). Transcriptome reveals insights into hepatic nutritional metabolism and gill immune responses adapted to cold stress in genetically improved farmed tilapia (GIFT: *Oreochromis niloticus*). Aquac. Rep..

[40] Grimble R.F., Grimble G.K. (1998). Immunonutrition: Role of sulfur amino acids, related amino acids, and polyamines. Nutrition.

[41] Isogai S., Takagi H. (2021). Enhancement of lysine biosynthesis confers high-temperature stress tolerance to *Escherichia coli* cells. Appl. Microbiol. Biotechnol..

[42] Zhu Y., Ding Q.L., Chan J., Chen P., Wang C.F. (2015). The effects of concurrent supplementation of dietary phytase, citric acid and vitamin D3 on growth and mineral utilization in juvenile yellow catfish *Pelteobagrus fulvidraco*. Aquaculture.

[43] Xu Y.Q., Huang J.H., Li W.F., Zheng Y.M., Jiang J., Ding Z.K. (2020). Dietary supplementation of vitamin E and citric acid could significantly promote the relative expression of PPARα and aconitase genes, concentration of polyunsaturated fatty acids, antioxidant enzyme activities, and growth of juvenile cobia. Aquaculture.

[44] Zhao S.F., Chen Z.C., Zheng J., Dai J.H., Ou W.H., Xu W.Q., Ai Q.H., Zhang W.B., Niu J., Mai K.S. (2019). Citric acid mitigates soybean meal induced inflammatory response and tight junction disruption by altering TLR signal transduction in the intestine of turbot, *Scophthalmus maximus* L. Fish Shellfish Immunol..

[45] Gracey A.Y., Fraser E.J., Li W., Fang Y., Taylor R.R., Rogers J., Brass A., Cossins A.R. (2004). Coping with cold: An integrative, multitissue analysis of the transcriptome of a poikilothermic vertebrate. Proc. Natl. Acad. Sci. USA.

[46] Li C.J., Liu X.Y., Hu C.C., Yan J.J., Qu Y.F., Li H., Zhou K.Y., Li P. (2025). Genome-wide characterization of the TRP gene family and transcriptional expression profiles under different temperatures in gecko *Hemiphyllodactylus yunnanensis*. Comp. Biochem. Physiol. Part D Genom. Proteom..

[47] Chang J., Pan Y.F., Liu W.T., Xie Y., Hao W.Y., Xu P., Wang Y.H. (2022). Acute temperature adaptation mechanisms in the native reptile species *Eremias argus*. Sci. Total Environ..

[48] Luo B.W., Gan W., Liu Z.W., Shen Z.G., Wang J.S., Shi R.C., Liu Y.Q., Liu Y., Jiang M., Zhang Z.R. (2016). Erythropoeitin Signaling in Macrophages Promotes Dying Cell Clearance and Immune Tolerance. Immunity.

[49] Accili D., Arden K.C. (2004). FoxOs at the Crossroads of Cellular Metabolism, Differentiation, and Transformation. Cell.

[50] Ge G.D., Long Y., Shi L.Y., Ren J., Yan J.J., Li C.T., Li Q., Cui Z.B. (2020). Transcriptomic profiling revealed key signaling pathways for cold tolerance and acclimation of two carp species. BMC Genom..

[51] Ma F., Zhao L., Ma R.L., Wang J., Du L.Q. (2023). FoxO signaling and mitochondria-related apoptosis pathways mediate tsinling lenok trout (*Brachymystax lenok tsinlingensis*) liver injury under high temperature stress. Int. J. Biol. Macromol..

[52] Ji L.Q., Shi Q., Shangguan Y.S., Chen C., Zhu J.X., Dong Z., Hong X.Y., Liu X.L., Wei C.Q., Zhu X.P. (2025). Molecular Response and Metabolic Reprogramming of the Spleen Coping with Cold Stress in the Chinese Soft-Shelled Turtle (*Pelodiscus sinensis*). Antioxidants.

[53] Xu D.D., You Q.C., Chi C.F., Luo S.Y., Song H.B., Lou B., Takeuchi Y. (2018). Transcriptional response to low temperature in the yellow drum (*Nibea albiflora*) and identification of genes related to cold stress. Comp. Biochem. Physiol. Part D Genom. Proteom..

[54] Wang M.Y., Xu G.C., Tang Y.K., Xu P. (2020). Transcriptome analysis of the brain provides insights into the regulatory mechanism for *Coilia nasus* migration. BMC Genom..

[55] Su J., Li W.H., Li H.Q., Zhou Z.X., Miao Y., Yuan Y., Li Y.C., Tao M., Zhang C., Zhou Y. (2024). Comparative transcriptomic analysis of the brain-liver Axis reveals molecular mechanisms underlying acute cold stress response in Gynogenetic Mrigal carp. Aquaculture.

[56] OuYang J.H., Gao Y.C., Wei Y.F., Huang H.P., Ge Y., Zhao J., Gong S.P. (2021). Transcriptome analysis reveals reduced immunity and metabolic level under cold stress in *Mauremys mutica*. Front. Mar. Sci..

[57] Tripathi A., Debelius J., Brenner D.A., Karin M., Loomba R., Schnabl B., Knight R. (2018). The gut–liver axis and the intersection with the microbiome. Nat. Rev. Gastroenterol. Hepatol..

[58] Fan J., Wang D.X. (2024). Serum uric acid and nonalcoholic fatty liver disease. Front. Endocrinol..

[59] Zhang H., Xiu M., Li H.L., Li M.C., Xue X.M., He Y.W., Sun W.Y., Yuan X., Liu Z., Li X. (2023). Cadmium exposure dysregulates purine metabolism and homeostasis across the gut-liver axis in a mouse model. Ecotoxicol. Env. Saf..

[60] Toledo-Ibelles P., Gutiérrez-Vidal R., Calixto-Tlacomulco S., Delgado-Coello B., Mas-Oliva J. (2021). Hepatic Accumulation of Hypoxanthine: A Link Between Hyperuricemia and Nonalcoholic Fatty Liver Disease. Arch. Med. Res..

[61] Thumser A.E., Voysey J.E., Wilton D.C. (1994). The binding of lysophospholipids to rat liver fatty acid-binding protein and albumin. Biochem. J..

[62] Zhang X., Lau H.C., Ha S., Liu C.F., Liang C., Lee H.W., Ng Q.W., Zhao Y., Ji F.F., Zhou Y.F. (2025). Intestinal TM6SF2 protects against metabolic dysfunction-associated steatohepatitis through the gut–liver axis. Nat. Metab..

