# Supplementary material for: The Influence of Acute Cold Stress on Intestinal Health of the Juvenile Chinese Soft-Shelled Turtle (Pelodiscus sinensis)
Source: Animals (Basel). 2026 Jan 14;16(2):256. doi: 10.3390/ani16020256 (PMC12837940; doi:10.3390/ani16020256)
Supplement: Supplementary file 1 [file animals-16-00256-s001.zip › animals-4038305-supplementary.pdf]

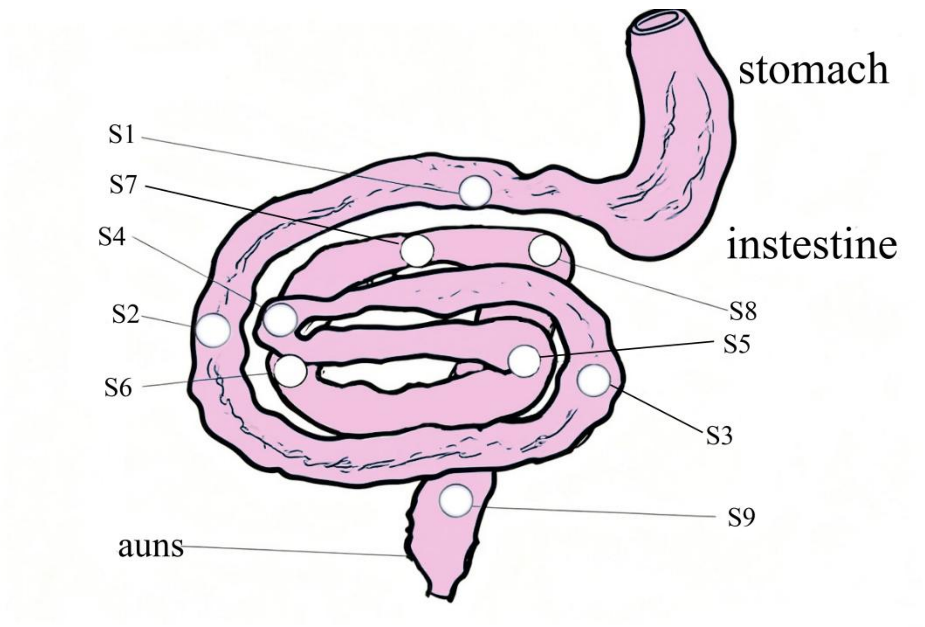

Figure S1. Sketch of the Intestine division. Nine segments were sampled longitudinally in the Chinese soft-shelled turtle intestine, and labeled as S1,S2, S3, S4, S5, S6, S7, S8 and S9, respectively[1]. In this experiment, the regions from S1 to S5 was selected to histological and micorbiota.

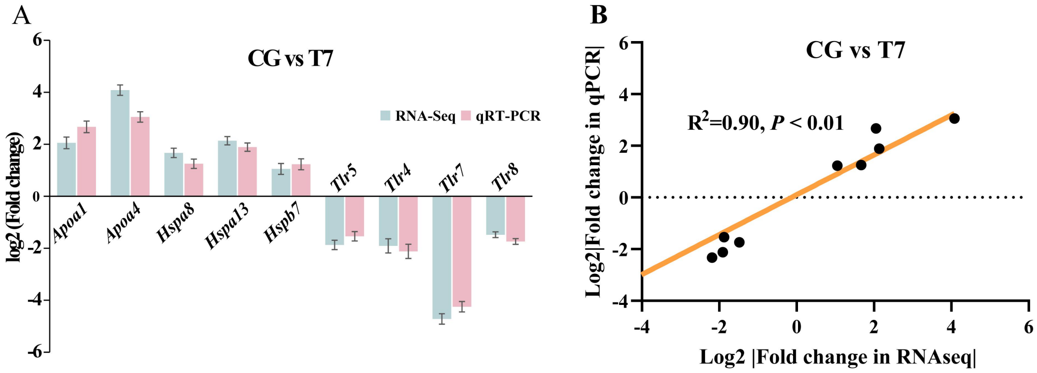

Figure S2. Pearson correlation analysis of mRNA expression levels of random 9 genes between RNA-seq and real-time PCR results in CG and T7. “CG” indicated the control group. “T7” indicated 7 °C cold stress group.

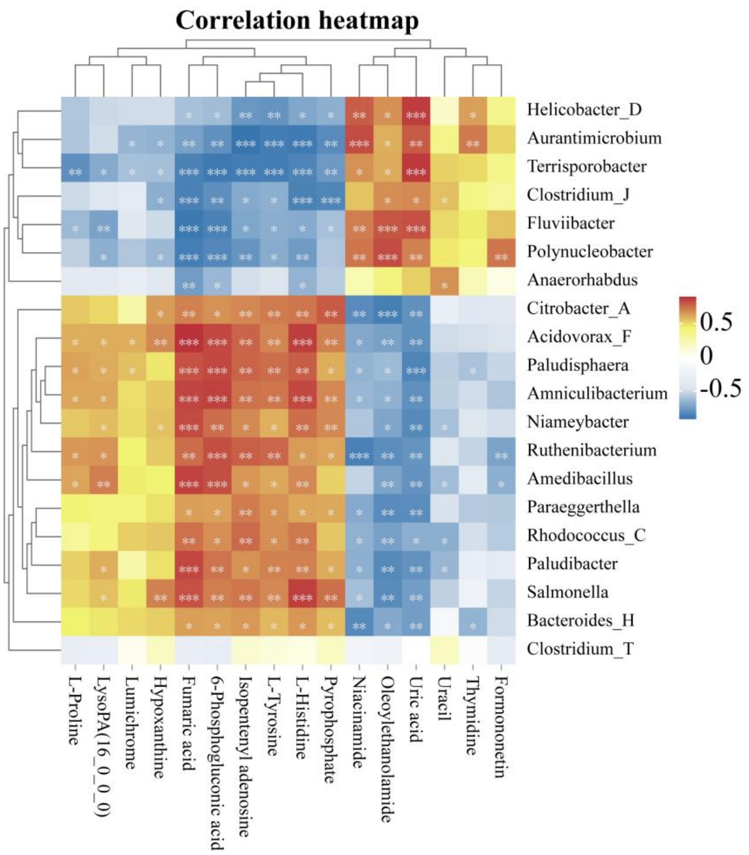

Figure S3. Spearman correlation analysis between the top 20 intestinal microbiota by abundance and liver metabolites. *indicates P < 0.05, **indicates P < 0.01, **indicates P < 0.001.

Table S1. The qRT-PCR primer sequences of the Chinese soft-shelled turtle.

Gene	Forward sequences (5’→3’)	Reverse sequences (5’→3’)
β-actin	AGACCCGACAGACTACCTCA	CACCTGACCATCAGGCAACT
Tlr4	TGCTGGCATTCTGTTCG	GGCATCGTAGGTGTCGTTC
Tlr5	TCTCACTGTTCATCTTCA	TATCTATTGCTTGCTTACG
Tlr7	CTTGTCAGGTAATGCTAT	TGTTATCACTCAGGTCTA
Tlr8	GAAGTCGTTCCACAGATAC	CCATACATGCTACACTGAG
Hspb7	AGCCTGTTCGGTGAGGACTTC	CTCTTTTTGGAGGCATGGG
Apoa1	GCTGGCTCCCTACTACACGC	CAGGACCTCCATCTTCTGCTTG
Apoa4	CTGACATCAGCAAGCAGCTC	TGGATCTGCTCCTTCAGCTT
Hspa8	CGTTGCCTTCACAGATAC	CGTGTTGGTAGGATTCATT
Hspa13	ACTCTACACCATTCTTCTC	TTCAACACCTGCTCAATA

Table S2. Overview of the transcriptomic sequencing quality in the CG and T7.

Sample	Raw Reads	Raw Bases	Clean Reads	Clean Data	Clean Reads%	Q20%	Q30%	GC content %	TMG%
CG1	45259474	6.83G	44343964	6.67G	97.98	98.70	96.46	48.54	83.85

CG2	52313480	7.90G	51369346	7.73G	98.20	98.79	96.68	48.53	83.83
CG3	58108660	8.77G	56907382	8.57G	97.93	98.69	96.40	48.52	83.76
T71	48895338	7.38G	47912828	7.21G	97.99	98.70	96.46	48.51	82.64
T72	58077480	8.77G	56900246	8.56G	97.97	98.70	96.47	48.64	82.49
T73	50993552	7.70G	49970232	7.51G	97.99	98.69	96.46	48.83	82.03

"CG" indicated the control group. "T7" indicated 7 ℃ cold stress group. "TMG" indicated the ratio of the total clean reads mapped to genome.

Table S3. The DEGs enriched in immune pathways of the intestines in CG and T7.

Pathway	DEGs	Name of DEGs
Cytokine-cytokine receptor interaction	41	*Edar*↑, *Il17b*↑, *Epor*↑, *Cntf*↑, *Tnfrsf13b*↑, *Il18rap*↑, *Bmp7*↓, *Ccr4*↓, *Tnfsf10*↓, *Inhba*↓, *Cd40lg*↓, *Il12rb1*↓, *Ccr7*↓, *Csf1r*↓, *Tnfsf8*↓, *Il2rb*↓, *Eda2r*↓, *Cxcr3*↓, *Il1Rl1*↓, *Ifnlr1*↓, *Cx3cr1*↓, *Ngf*↓, *Il23r*↓, *Tnfrsf19*↓, *Tnf*↓, *Il18r1*↓, *Il7R*↓, *Il26*↓, *Tnfrsf18*↓, *Tnfrsf17*↓, *Tnfrsf4*↓, *Bmpr1b*↓, *Ccr6*↓, *Nfrsf8*↓, *Tnfrsf11a*↓, *Il22*↓, *Tnfsf11*↓, *Csf3r*↓, *Bmp6*↓, *Cxcr5*↓, *Tnfsf14*↓
FoxO signaling pathway	28	*Bnip3*↑, *Bcl6*↑, *Mapk13*↑, *Irs4*↑, *Kras*↑, *Prkab*↑, *Mapk11*↑, *Smad4*↑, *Gadd45a*↑, *Cdkn1a*↑, *Ccng2*↑, *Foxo4*↑, *Pik3ca*↑, *Hras*↑, *Bcl2L11*↑, *Braf*↑, *Mdm2*↑, *Igf1*↑, *Plk2*↓, *Irs1*↓, *S1pr4*↓, *Egf*↓, *Ccnd1*↓, *Tnfsf10*↓, *G6pc2*↓, *Atm*↓, *Homer2*↓, *Ccnd2*↓, *Il7r*↓
Cellular senescence	24	*Serpine1*↑, *Mapk13*↑, *Kras*↑, *Mapk11*↑, *Sqstm1*↑, *Cdkn1a*↑, *Lin9*↑, *Gadd45a*↑, *Mdm2*↑, *Hras*↑, *Pik3ca*↑, *Smad2*↓, *Myc*↓, *Atr*↓, *Map2k3*↓, *Rbl1*↓, *Ccnd1*↓, *Nfatc2*↓, *Atm*↓, *Cdk6*↓, *Chek2*↓, *Zfp36*↓, *Ccnd2*↓, *Ets1*↓
Apoptosis	22	*Tuba1c*↑, *Ctsz*↑, *Ctsb*↑, *Kras*↑, *Hras*↑, *Pik3ca*↑, *Bcl2l11*↑, *Gadd45a*↑, *Pmaip1*↑, *Ddit3*↑, *Fos*↑, *Traf2*↓, *Tnfsf10*↓, *Ctso*↓, *Tnf*↓, *Bcl2a1*↓, *Ngf*↓, *Tradd*↓, *Casp2*↓, *Atm*↓, *Map3k14*↓, *Dab2ip*↓
Cell adhesion molecules	20	*Cd274*↑, *Cldn7*↑, *Nectin3*↑, *Vsir*↓, *Cd28*↓, *Cadm1*↓, *Cd40lg*↓, *Cldn18*↓, *Ptprc*↓, *Sele*↓, *Nlgn1*↓, *Ntng1*↓, *Vcam1*↓, *Cldn10*↓, *Itgal*↓, *Cd6*↓, *Cd2*↓, *Cd8b*↓, *Siglec1*↓, *Nlgn4x*↓
NOD-like receptor signaling pathway	19	*Ctsb*↑, *Mapk11*↑, *Mapk13*↑, *Txnip*↑, *Panx1*↑, *Ticam1*↑, *Gprc6a*↑, *Tnfaip3*↑, *Map1Lc3a*↑, *Traf6*↓, *Traf2*↓, *Tlr4*↓, *Casr*↓, *Tnf*↓, *Rnasel*↓, *Pstpip1*↓, *Plcb2*↓, *Atg5*↓, *Mfn2*↓
p53 signaling pathway	16	*Serpine1*↑, *Sesn2*↑, *Ccng2*↑, *Igf1*↑, *Mdm2*↑, *Gadd45a*↑, *Pmaip1*↑, *Cdkn1a*↑, *Sesn1*↓, *Ccng1*↓, *Ccnd1*↓, *Atr*↓, *Ccnd2*↓, *Chek2*↓, *Cdk6*↓, *Atm*↓
Toll-like receptor signaling pathway	14	*Mapk13*↑, *Fos*↑, *Pik3ca*↑, *Ticam1*↑, *Mapk11*↑, *Map3k8*↑, *Tnf*↓, *Tlr8*↓, *Tlr5*↓, *Traf6*↓, *Tlr7*↓, *Tlr2*↓, *Tlr4*↓, *Map2k3*↓
Herpes simplex virus 1 infection	14	*Pik3ca*↑, *Ticam1*↑, *Socs3*↑, *Tlr2*↓, *Zfp90*↓, *Srsf1*↓, *Tnfsf14*↓, *Traf2*↓, *Traf6*↓, *Rnasel*↓, *Tradd*↓, *Srsf6*↓, *C5*↓, *Tnf*↓
PPAR signaling pathway	12	*Apoa1*↑, *Fabp2*↑, *Fabp6*↑, *Scd*↑, *Apoa2*↑, *Acsbg2*↑, *Cd36*↑, *Fabp1*↑, *Plin2*↑, *Acox3*↓, *Fads2*↓, *Hmgcs1*↓

"CG" indicated the control group. "T7" indicated 7 ℃ cold stress group. "DEGs" indicated differential expressed genes.

Reference

1. Zhang, Z.B.; Song, R.X.; Xing, X.X.; Wang, L.W.; Niu, C.J. Division of Chinese soft-shelled turtle intestine with molecular markers is slightly different from the morphological and histological observation. *Integr Zool* **2018**, *13*, 112-121.
